# Myeloproliferative Neoplasm or Reactive Process? A Rare Case of Acute Myeloid Leukemia and Transient Posttreatment Megakaryocytic Hyperplasia with JAK-2 Mutation

**DOI:** 10.1155/2016/6054017

**Published:** 2016-09-26

**Authors:** Steven Wang, Jie Yan, Guangde Zhou, Rebecca Heintzelman, J. Steve Hou

**Affiliations:** Department of Pathology and Laboratory Medicine, Drexel University College of Medicine, 245 N. 15th Street, MS 435, Philadelphia, PA 19102, USA

## Abstract

Myeloproliferative neoplasms (MPNs) are hematopoietic malignancies characterized by unchecked proliferation of differentiated myeloid cells. The most common BCR-ABL1-negative MPNs are polycythemia vera, essential thrombocythemia, and primary myelofibrosis. The discovery of JAK2 V617F mutation has improved our understanding of the molecular basis of MPN. The high frequency of JAK2 mutation in MPN makes JAK2 mutation testing an essential diagnostic tool and potential therapeutic target for MPN. Here, we present a rare case of a 34-year-old patient who was initially diagnosed with acute myeloid leukemia (AML) with mutated NPM1. After chemotherapy treatment followed by granulocyte colony stimulating factor administration, the patient achieved complete remission of AML. However, the bone marrow showed hypercellularity with granulocytic hyperplasia, markedly increased atypical megakaryocytes (50.2/HPF) with focal clustering, and reticulin fibrosis (3/4). JAK2 V617F mutation was also detected. Considering the possibility of AML transformed from a previous undiagnosed MPN, patient underwent peripheral blood allogenic stem cell transplant. This case illustrates the diagnostic challenges of firmly establishing a diagnosis between similar, but distinct, disease entities and an accurate clinicopathological differentiation is crucial.

## 1. Introduction

Myeloproliferative neoplasms (MPNs) are characterized by excessive production of terminally differentiated myeloid cells. The most common BCR-ABL1-negative MPNs are polycythemia vera (PV), essential thrombocythemia (ET), and primary myelofibrosis (PMF). In MPN, the JAK2 V617F mutation is the most prevalent mutation discovered in 2005 [1]. Almost all patients with PV harbor a JAK2 mutation that includes JAK2 V617F in about 97% of the patients. JAK2 V617F also occurs in approximately 40–60% of patients with ET or PMF [1–4].

MPNs have a risk of transforming to secondary acute myeloid leukemia (sAML). Leukemic transformation of ET, PV, and PMF occurs at rates of approximately 1%, 4%, and 20%, respectively, over the first decade from time of MPN chronic phase diagnosis. After leukemic transformation, patients have poor prognosis with an adverse outcome within a few months. The frequency of JAK2 mutation in de novo AML is about 1%. In contrast, the prevalence of JAK2 mutation in AML transformed from MPN is about 50% [5–7].

Administration of granulocyte macrophage colony stimulating factor (GM-CSF) in combination with thrombopoietin (TPO) to patients who had received chemotherapy for acute myeloid leukemia (AML) has been found to cause bone marrow hypercellularity, marked megakaryocytic hyperplasia, megakaryocytic atypia, and reticulin fibrosis with a rapid resolution of the morphologic abnormalities after discontinuation of TPO. These findings, in combination with a possible peripheral blood leukoerythroblastic reaction, yield an overall picture that morphologically resembles that seen in patients with MPN [8, 9].

Here, we present a rare case of a patient with a quite complicated clinical course and histopathologic picture of megakaryocytic hyperplasia following treatment and resolution of acute myeloid leukemia. This case illustrates the diagnostic challenges of firmly establishing a diagnosis between similar, but distinct, disease entities.

## 2. Case Presentation

**Figure 1:**
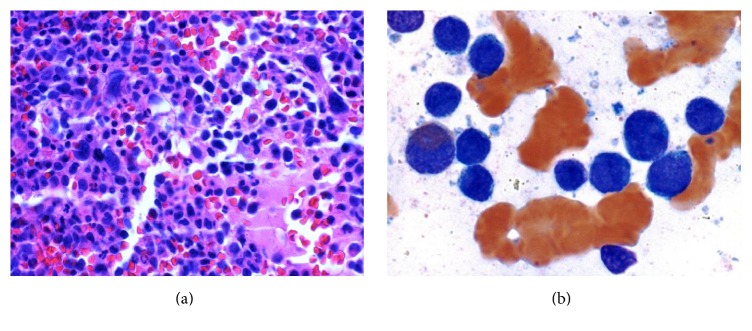
(a) First bone marrow biopsy showing hypercellular marrow packed with excess of immature myeloid cells and nearly absence of megakaryocytes. (b) First bone marrow aspirate showing numerous myeloblasts.

Patient was a 34-year-old male with a medical history significant for gout, chronic pain secondary to a motor vehicle accident, and bipolar disorder. There was no documented prior history of childhood malignancy or radiation. He had a gout exacerbation which was followed by the development of fever, sweats, and sore throat. He presented to a local urgent care center where he was found to have moderate splenomegaly and significantly high white blood cell (WBC) count with circulating blasts in the peripheral blood (Table 1). The first bone marrow (BM) biopsy performed showed 95% cellularity with 70% CD13, CD33, CD117, HLA-DR(+), and CD34(−) myeloblasts demonstrating Sudan black B positivity (Figure 1). Cytogenetic and molecular studies revealed a normal male karyotype with NPM1 mutation. Based on these findings and the fact that this patient has no known history of chronic myeloproliferative disease, a diagnosis of AML with mutated NPM1 was made and induction chemotherapy with FLAG-IDA regimen (fludarabine, cytarabine, idarubicin, and granulocyte colony stimulating factor) was started and GM-CSF was given from day 6 of the induction. The patient achieved complete remission after induction therapy.

**Figure 2:**
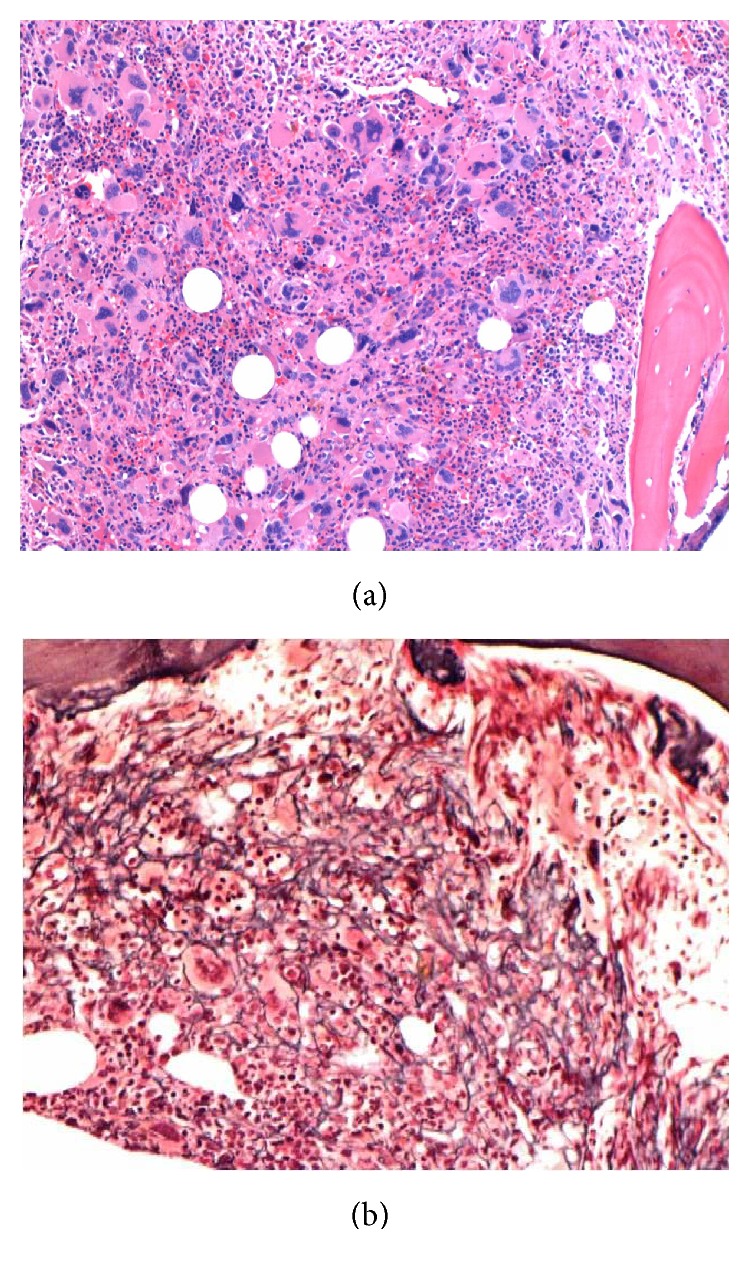
(a) Postinduction bone marrow biopsy showing 95% cellularity with granulocytic hyperplasia and numerous large, hyperlobulated megakaryocytes with clustering. (b) Reticulin stain of the postinduction bone marrow showing significantly increased reticulin fibrosis (3/4).

The postinduction bone marrow biopsy was performed and showed 95% cellularity with granulocytic hyperplasia, markedly increased megakaryocytes (50.2/HPF), and increased reticulin fibrosis (3/4). No blasts were identified in the marrow. The megakaryocytes were large, hyperlobulated, and clustered (Figure 2). The peripheral platelet count was also dramatically increased (Table 1). Given the histologic and clinical pictures, the possibility of an underlying MPN was considered and supported by positive JAK2 V617F mutation. In retrospect of note, his blood count from July 2013 showed a platelet count of 748,000/*μ*L with a normal white count and hemoglobin level.

**Figure 3:**
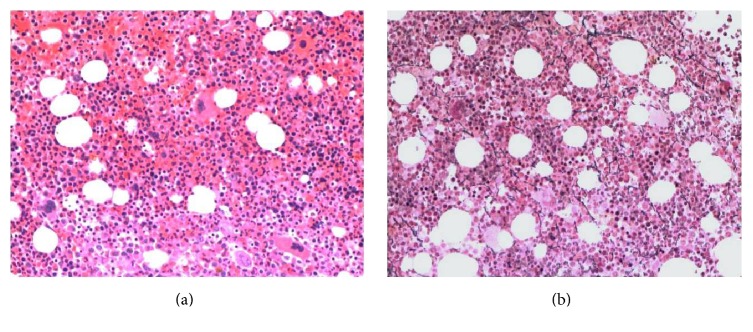
(a) Postconsolidation bone marrow biopsy showing 80% cellular marrow and mild megakaryocytic hyperplasia. The scattered megakaryocytes are morphologically unremarkable. (b) Reticulin stain of the postconsolidation bone marrow showing complete resolution of marrow fibrosis.

The patient started consolidation chemotherapy with high dose intermittent ARA-C (HiDAC). The subsequent bone marrow biopsy revealed 80% cellular marrow with mild megakaryocytic hyperplasia (6.7/HPF) and complete resolution of marrow fibrosis with no blasts identified in the marrow (Figure 3). The megakaryocytes were morphologically unremarkable with focal clustering. The peripheral platelet count was also within the normal range (Table 1). The JAK2 V617F mutation test was repeated and remained positive.

Considering the possibility of AML transformed from a previous undiagnosed MPN, patient underwent peripheral blood allogenic stem cell transplant from his HLA-match, ABO mismatch sister after myeloablation. Day 30 chimerism revealed 100% donor myeloid cells and 64% donor T cells and a day 60 evaluation for chimerism showed 100% donor myeloid cells and 99% donor T cells. The follow-up bone marrow biopsy revealed normocellular marrow with no overt phenotypic abnormalities. The megakaryocytes were mildly decreased in number and morphologically unremarkable without clustering. His hemoglobin level was 12.3 g/dL, platelet count 93,000/*μ*L, and WBC count 2,000/*μ*L with normal differentiation. The JAK2 V617F mutation was not detected. The patient was last seen in July 2016. He remained in complete remission with no clinical or laboratory evidence of relapse of AML and MPN.

## 3. Discussion

PV is characterized by unregulated red blood cell overproduction and ET by excessive platelet production, while PMF is a clonal stem cell disorder with a consequence of megakaryocytic and granulocytic proliferation, progressive marrow fibrosis, and extramedullary hematopoiesis [1–3].

The JAK family proteins (JAKs) are cytoplasmic tyrosine kinases that play important roles in cytokine receptor superfamily signaling which are crucial for normal hematopoiesis. Aberrant hyperactivation of JAK signaling pathway has increasingly been implicated in the pathogenesis of hematopoietic malignancies [1, 4, 5]. The JAK2 V617F mutation leads to a constitutively active JAK2 kinase signaling by abrogating the negative regulatory activity of the pseudokinase domain JH2 of the encoded JAK2 tyrosine kinase. High frequency of JAK2 mutation in MPN makes JAK2 mutation testing a frontline screen for highly suspected MPN. Quantitative JAK2 mutation testing can not only provide prognostic information about MPN and monitor minimal residual disease after stem cell transplant for JAK2-positive patients with myelofibrosis but also be used to monitor disease progression and response to therapy [10–12]. The JAK2 inhibitor ruxolitinib was approved for the treatment of MF and more recently for the treatment of PV with inadequate response or intolerant adverse effects to hydroxyurea. Numerous other JAK2 inhibitors are currently in phase II/III clinical trials. In phase III RESPONSE trial in PV patients, ruxolitinib was proved to be the best available therapy in improving symptoms, reducing leukocyte and platelet counts, and reducing hematocrit as well as spleen size after 32 weeks of treatment. However, a subset of patients will probably develop resistance to JAK2 inhibitor therapy, possibly through the emergence of resistance mutations [13–16].

AML is a clonal hematopoietic disorder involving clonal hematopoietic stem cell or a lineage-specific progenitor cell, resulting in the accumulation of blast cells associated with disrupted granulomonocytic differentiation in the bone marrow. AML accounts for approximately 80% of all adult leukemia cases with the majority of the cases arising de novo [6, 7]. Therapy-related AML (t-AML) occurs as late complications of cytotoxic chemotherapy and/or radiation therapy administered for a prior neoplastic or nonneoplastic disorder. Secondary AML (sAML) can develop as a consequence of preexisting myelodysplastic syndrome (MDS) or MPN [17–20]. Both tAML and sAML are associated with unfavorable chromosomal abnormalities and worse prognosis as compared with de novo AML. Patients with MPN are at an elevated risk for leukemic transformation and exhibit significantly more chromosomal aberrations after transformation. The underlying mechanism of AML transformation from a preexisting MPN is not well understood. Several genes have been repeatedly identified to be mutated in the MPN blast phase, such as TET2, IDH1, IDH2, IKZF1, and RUNX1 [7, 18]. Recently, mutations in the serine/arginine-rich splicing factor 2 (SRSF2) gene were found to be present in 18.9% of patients with sAML versus 5.6% of patients with de novo AML. TP53 mutations were also reported to be an independent prognostic factor for poor survival in sAML [18–20]. Additional study demonstrated that more than 50% of JAK2 mutation positive MPN transforms into JAK2 mutation negative AML and is associated with much shorter interval between MPN diagnosis and leukemia transformation (3 versus 10 years) [17–20]. The prognostic implication of JAK2 V617F status in this setting remains debatable.

JAK2 mutation has been found in approximately 1% of de novo AML patients, more commonly in t(8; 21) AML. In addition, activating JAK2 gene fusions with the TEL (ETV6) (TEL-JAK2) and PCM1 genes has been identified in leukemia patients. A recent study from a large cohort of patients with AML (*n* = 77) demonstrated elevated levels of activated phospho-Jak2 (p-JAK2) in bone marrow samples which is associated with high white blood cell count, low platelet count, and shorter survival in patients with either de novo or secondary AML. In this study, JAK2 V617F mutation was detected in only 1 of the 77 patients, indicating that alternative mechanisms account for JAK2 signal activation and may represent a potential therapeutic target [6, 20, 21].

Administration of granulocyte macrophage colony stimulating factor (GM-CSF) and thrombopoietin (TPO) to patients treated with chemotherapy for AML has been found to cause bone marrow hypercellularity, marked megakaryocytic hyperplasia, megakaryocytic atypia, and reticulin fibrosis with a rapid resolution of the morphologic abnormalities after discontinuation of TPO [8, 9]. In this case, the patient received GM-CSF only after induction therapy. The bone marrow changes could be due to a reactive process occurring after chemotherapy for AML caused by GM-CSF administration. This is supported by the findings of resolving megakaryocytic hyperplasia and reticulin fibrosis with normalization of the platelet count on the postconsolidation bone marrow after cessation of administration of GM-CSF. However, data in animal models and clinical trials have shown that, upon receiving GM-CSF plus TPO, the florid megakaryocytic hyperplasia and reticulin fibrosis in the bone marrow can be attributed primarily to TPO administration with minimal contribution from the GM-CSF [22–25].

In this patient, the diagnosis of de novo AML with mutated NPM1 was made on his first bone marrow biopsy showing 70% CD13, CD33, CD117, HLA-DR(+), and CD34(−) myeloblasts with circulating blasts and marked leukocytosis. After induction therapy, the patient achieved complete remission of AML. However, the bone marrow showed hypercellularity with granulocytic hyperplasia, markedly increased atypical megakaryocytes (50.2/HPF) with clustering, and reticulin fibrosis (3/4). The peripheral platelet count was also dramatically elevated. The possibility of sAML transformed from a preexisting undiagnosed MPN was considered and supported by presence of persistent JAK2 V617F mutation, history of long standing gout, and marked thrombocytosis approximately 2 months before his AML presentation. As well documented, after leukemic transformation from MPN, patients have poor prognosis with an adverse outcome within a few months. In this patient, the absence of a complex cytogenetic abnormality, complete remission of AML after induction chemotherapy, and rapid normalization of bone marrow after consolidation all argue against the diagnosis of sAML transformation from preexisting MPN. It is less likely that this is a de novo AML with JAK2 mutation given the frequency of about 1% JAK2 mutation in de novo AML and about 50% in AML transformed from MPN. All these facts also raise the possibility that there might be two different hematopoietic clones present in the bone marrow with full-blown manifestations during the course of his disease. Quantitative analysis of the variant allele frequency for JAK2 and NPM1 mutation might be helpful to solve the diagnostic dilemma.

This rare case demonstrates the challenges of firmly establishing a diagnosis between similar, but distinct, disease entities. Synthesized interpretation of available data and close clinical follow-up are required.

## Figures and Tables

**Table 1:** Pertinent peripheral blood findings.

Time of blood collection	White blood cell count	Hemoglobin	Platelet count
First bone marrow biopsy	46,000/*μ*L with blasts	10.5 g/dL	210,000/*μ*L
Postinduction chemotherapy	7,600/*μ*L with normal differentiation	11.7 g/dL	731,000/*μ*L
Postconsolidation chemotherapy	8,000/*μ*L with normal differentiation	14.1 g/dL	358,000/*μ*L
